# Supplementary material for: Stratification-based Instrumental Variable Analysis Framework for Nonlinear Effect Analysis
Source: Biostatistics. Author manuscript; Available in PMC 2026 Jan 21. (PMC12665183; doi:10.1093/biostatistics/kxaf043)
Supplement: Supplementary material [file EMS211762-supplement-Supplementary_material.pdf]

Stratification-based Instrumental Variable Analysis Framework for Nonlinear Effect Analysis
Supplementary Materials

Haodong Tian[1,2,*], Ashish Patel[3], Stephen Burgess[3,4]

[1] Center for Genomic Medicine, Massachusetts General Hospital
[2] Broad Institute of MIT and Harvard
[3] MRC Biostatistics Unit, University of Cambridge
[4] BHF Cardiovascular Epidemiology Unit, University of Cambridge
[*]htian2@mgh.harvard.edu

Abstract

This supplementary material provides additional details to facilitate a better understanding of the main manuscript. It includes algorithmic descriptions, application examples, formula derivations, inference procedures, relevant discussions, and additional simulations and results.

Contents

Appendix A: Algorithm of the stratification methods **2**

Appendix B: Potential use cases under the rank-preserving assumption **3**

Appendix C: Rank preserving assumption in *Angrist&Krueger (1991)* **4**

Appendix D: Scalar-on-function model properties **6**

Appendix E: Linearity testing **8**

Appendix F: Complicated model scenario and their model properties **8**

Appendix G: Complete algorithm of the SSS framework **11**

Appendix H: Supplementary simulation **12**

Appendix I: UK Biobank real application with females **15**

Appendix J: Supplementary results of the real application **16**

©2025 Tian, Patel, and Burgess.

License: CC-BY 4.0, see https://creativecommons.org/licenses/by/4.0/.

Appendix A: Algorithm of the stratification methods

Algorithm 1: Prediction-based stratification (residual stratification)

Assumption: $X = t[f(Z) + g_X(U, \epsilon_X)]$
Input: The individual-level data $(Z_i, X_i, Y_i)_{i=1,\dots,n}$
$K \leftarrow$ the number of strata
$t[\cdot] \leftarrow$ the monotone bijective function for Z-X relationship, or $t[x] \equiv x$
if $f(\cdot)$ *is known* **then**
    $e_i \leftarrow t^{-1}[X_i] - f(Z_i)$; obtain individual residuals
else
    Let $f(z) = \theta_1 f_1(z) + \theta_2 f_2(z) + \cdots + \theta_L f_L(z)$
    Model selection based on $\{t^{-1}[X], Z, \{\hat{\theta}_l, f_l(Z)\}_{l=1,\dots,L}\}$ via, e.g., BIC or cross validation
    $\tilde{f}(z) \leftarrow$ the best model
    $e_i \leftarrow t^{-1}[X_i] - \tilde{f}(Z_i)$; obtain individual residuals
$\{e_i^*\} \leftarrow$ rank $\{e_i\}$
for $k \leftarrow 1$ **to** K **do**
    $\mathcal{P}_k \leftarrow$ the individual index of the $[(k-1)\lfloor \frac{n}{K} \rfloor + (1 : \lfloor \frac{n}{K} \rfloor)]$-th ranked individual in $\{e_i^*\}$
    $\mathcal{S}_k \leftarrow$ the individual data set (Z_i, X_i, Y_i) with index $\mathcal{P}_k$
Output: $\mathcal{S}_k, k = 1, \dots, K$

Algorithm 2: Matching-based stratification (doubly-ranked stratification)

Assumption: rank-preserving assumption between the instrument and exposure
Input: the individual-level data $(Z_i, X_i, Y_i)_{i=1,\dots,n}$
$K \leftarrow$ the number of strata
$S \leftarrow$ the size of pre-strata, an integer multiple of K. By default, $S = K$
$\{Z_i^*\} \leftarrow$ rank $\{Z_i\}$
for $s \leftarrow 1$ **to** $\lfloor \frac{n}{S} \rfloor$ **do**
    $\mathcal{P}_s \leftarrow$ the individual index of the $[(s-1)S + (1 : S)]$-th ranked individual in $\{Z_i^*\}$
    $\{X_i^*; i \in \mathcal{P}_s\} \leftarrow$ rank $\{X_i; i \in \mathcal{P}_s\}$
for $k \leftarrow 1$ **to** K **do**
    $\mathcal{S}_k \leftarrow$ the set of (Z_i, X_i, Y_i) with individual corresponding to the $[(k-1)\frac{S}{K} + (1 : \frac{S}{K})]$-th individual in $\{X_i^*; i \in \mathcal{P}_s\}$ for all s
Output: $\mathcal{S}_k, k = 1, \dots, K$

Appendix B: Potential use cases under the rank-preserving assumption

We list some potential use cases and discuss the plausibility of the rank-preserving assumption in some examples.

Example 1 *Inspired by Angrist and Krueger (1991), consider a country where children born in different months of the year start school at the same calendar time but with different ages. Additionally, compulsory schooling laws mandate that students must remain in school until a specific birthday (e.g., 16th or 17th). In this case, birth time or season could serve as an instrument for schooling length, impacting outcomes such as earnings. Particularly, the rank-preserving assumption between birth time and schooling length is reasonable, as for any two individuals with the same birth time, the person with a longer schooling length should still maintain a longer schooling length, even if their birth times were counterfactually swapped with another identical birth time. We provided more explanation with the data of Angrist and Krueger (1991) in the next Appendix.*

Example 2 *Drawing from the Judge IV idea (Kling, 2006), strict judges and lenient judges may impose more or less prison time for the same criminal case. In many instances, the allocation of judges is random, making judge type a valid instrumental variable for analyzing the causal effect of prison time on various outcomes, such as income. Therefore, restricting the analysis to certain criminal cases, the rank-preserving assumption between judge type and prison time can be reasonable, as for specific case types, under any given judge, the person who serves a longer sentence in a pair will still serve longer.*

Example 3 *In Mendelian randomization, assume there exists a genetic variant or gene score that affects one phenotype of interest, such as weight or alcohol intake, for a group. Since the genetic variant can be reasonably considered to be randomly assigned, it can serve as an instrument for investigating the causal effect of the phenotype on an outcome of interest, such as cardiovascular disease. Given that the genetic effect follows a similar form (either constantly additive or multiplicative) within this group (e.g. male), the rank-preserving assumption between the gene and the phenotype may be plausible. This provides an opportunity to use the stratification approach to investigate the nonlinear causal effect.*

Appendix C: Rank preserving assumption in *Angrist&Krueger (1991)*

We provide a more detailed introduction to the rank-preserving assumption using real data from Angrist and Krueger (1991), where birth quarter is considered an instrument for years of education. The dataset, derived from the 1970 Census, consists of men born between 1920 and 1929. It includes 222,389 samples, each with recorded birth quarter (Q1–Q4) and years of education, which are rounded to the nearest integer (ranging from a minimum of 0 to a maximum of 18). The data is readily accessible via the R package `sketching`. We construct four groups, each corresponding to a birth quarter, and examine the distribution of years of education.

The distribution of years of education across birth quarters is presented in Figure 1, showing the proportion of individuals with each education level within each birth quarter group. The plot reveals a clear and consistent pattern: individuals born in later quarters tend to have fewer years of education. Specifically, for a given education level, the proportion of individuals with fewer years of education than that level generally decreases over the birth quarters Q1-Q4. This trend is expected due to the effect of birth quarter: among individuals who left school at a specific age, those born in later quarters typically have longer schooling durations than those born earlier in the same calender year. As a result, later birth quarters should have a lower proportion of individuals with fewer years of education and a higher proportion with more years of education. In this scenario, the effect of birth quarter on years of education appears relatively constant, supporting the rank-preserving assumption—namely, that the relative ordering of education lengths among individuals born in the same time period remains unchanged when their birth times shift. Although in this example, the instrument (birth quarter) is rounded to discrete values, its impact on doubly-ranked stratification should be minimal. This is because doubly-ranked stratification first aggregates similar instrument values, and the instrument's effect size is relatively small.

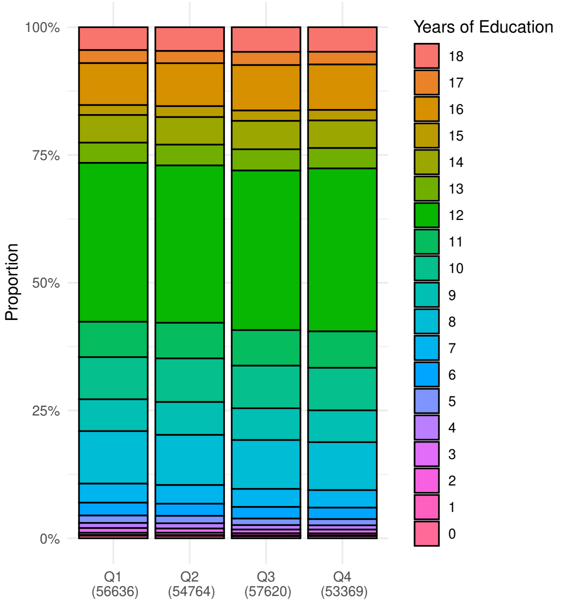

Figure 1: The education length distribution by quarter of birth (Q1-Q4) in the data of Angrist and Krueger (1991). The value in the brackets indicate the sample size in each quarter group.

The plausibly valid rank-preserving condition is the only additional requirement beyond the core assumptions of instrumental variables (IV) for nonlinear effect analysis in our SSS method. We proceed to analyze the data from Angrist and Krueger (1991) to investigate the effect of education

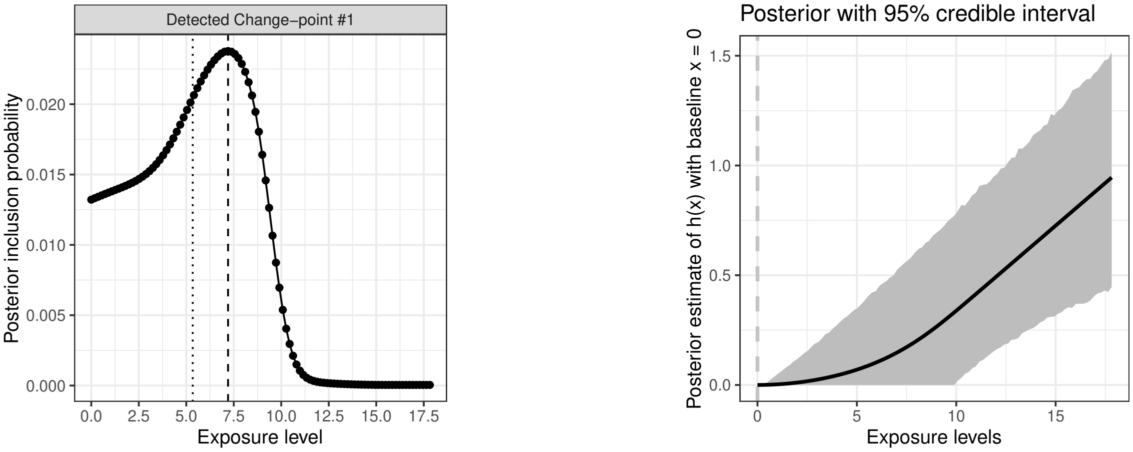

Figure 2: Nonlinear effect analysis results using the SSS method applied to the data from Angrist and Krueger (1991). The exposure variable is years of education, and the outcome is log weekly wages from the 1970 Census. Left panel: The posterior inclusion probability of the only detected change-point. The dashed and dotted lines represent the posterior mode and mean values, respectively. Right panel: The estimated effect function. The gray region represents the 95% credibility interval.

length on earnings (log weekly wages). Since the exposure (education length) is coarsened, we adopt the doubly-ranked stratification due to its proven ability to produce reliable results under coarsened exposure. For coarsened exposures, the number of strata should be carefully determined to avoid inducing excessive selection bias from measurement errors in the exposure (Tian et al., 2023). The optimal number of strata can be determined using the Gelman-Rubin statistics from the `DRMR` package, and in our example, we chose to set the number of strata to 10. We conducted the downstream analysis using our developed software, `SSS`.

There is one change-point detected by our methods, and the posterior inclusion probability (PIP) of this change-point is shown in the left panel of Figure 2. The predicted change-point value is 5.3 (posterior mean) and 7.2 (posterior mode). This change-point corresponds to the primary education level, typically 6 years of education, above which the effect of education length on earnings becomes evident. The estimated effect function, $h(x)$, with the baseline level at $x = 0$, is presented in the right panel of Figure 2. As shown, the effect of education length on earnings becomes significant after 10 years of education.

Appendix D: Scalar-on-function model properties

In this appendix we prove the scalar-on-function (SoF) model for the IV framework with stratification, and show the properties of the weight function.

Assume $Y = h(X) + U$ and the valid instrument Z. Assume that there exits the lower bound l for the exposure domain $\mathcal{X}$ such that $\mathbb{P}(l < X) = 1$ almost surely. We let the effect function is continuous. We have

$$\begin{aligned}
\frac{Cov(Z,Y)}{Cov(Z,X)} &= \frac{Cov(Z, h(X)+U)}{Cov(Z,X)} \\
&= \frac{Cov(Z, \int_l^X h'(x)\mathrm{d}x + h(l) + U)}{Cov(Z,X)} \\
&= \frac{Cov(Z, \int_l^\infty h'(x)I\{X \geq x\}\mathrm{d}x + h(l) + U)}{Cov(Z,X)} \\
&= \frac{Cov(Z, \int_l^\infty h'(x)I\{X \geq x\}\mathrm{d}x)}{Cov(Z,X)} \\
&= \int_{\mathcal{X}} h'(x) \underbrace{\frac{Cov(Z, I\{X \geq x\})}{Cov(Z,X)}}_{=:W(x)} \mathrm{d}x
\end{aligned} \tag{1}$$

Note that this conclusion is easily extended to the complex case $h(X, \epsilon)$ with exogenous term ϵ, given that $h(X,\epsilon) = \int_l^X h'(x,\epsilon)\mathrm{d}x + h(l,\epsilon)$ where $h'(x,\epsilon) = \delta h(x,\epsilon)/\delta x$ exists. In this case, we have $h'(x) = \mathbb{E}_\epsilon(h'(x,\epsilon))$ in Equation (1).

In terms of the weight function $W(x)$, we can show

$$\begin{aligned}
\int_{\mathcal{X}} W(x)\mathrm{d}x &= \frac{\int_{\mathcal{X}} Cov(Z, I\{X \geq x\})}{Cov(Z,X)} \\
&= \frac{Cov(Z, \int_{\mathcal{X}} I\{X \geq x\}\mathrm{d}x)}{Cov(Z,X)} \\
&= \frac{Cov(Z,X)}{Cov(Z,X)} = 1
\end{aligned} \tag{2}$$

Given the instrument-exposure model with linearity and homogeneity so that $X = \alpha Z + g_X(U, \epsilon_X)$ with confounder U and exogenous variable ϵ_X and w.o.l.g. the instrumental effect $\alpha > 0$. Denote $g_X(U, \epsilon_X)$ by E, we have

$$\begin{aligned}
W(x) &= \frac{[\mathbb{E}(Z|X \geq x) - \mathbb{E}(Z)]\mathbb{P}(X \geq x)}{Cov(Z,X)} \\
&= \frac{[\mathbb{E}(Z|X \geq x) - \mathbb{E}(Z|X < x)]\mathbb{P}(X < x)\mathbb{P}(X \geq x)}{Cov(Z,X)} \\
&= \frac{\mathbb{P}(X < x)\mathbb{P}(X \geq x)}{\alpha^2 Var(Z)} \left[\int_{\mathcal{Z}} z[f_Z(z|X \geq x) - f_Z(z|X \leq x)]\mathrm{d}z\right]
\end{aligned} \tag{3}$$

Since $f_Z(z|X \geq x) = \frac{f_Z(z)f_E(x-\alpha z)}{\mathbb{P}(X \leq x)}$, while $\mathbb{P}(E \geq x - \alpha z)$ is a monotone non-decreasing function over z; similar to $f_Z(z|X \leq x)$ with the monotone non-increasing function $\mathbb{P}(E \leq x - z)$; hence there exists an constant z^* such that $f_Z(z|X \geq x) \geq f_Z(z|X \leq x)$ when $z \geq z^*$ and $f_Z(z|X \geq x) \leq f_Z(z|X \leq x)$

otherwise. Therefore,

$$
\begin{aligned}
\int_{\mathcal{Z}} z[f_Z(z|X \geq x) - f_Z(z|X \leq x)]\mathrm{d}z = &\int_{\mathcal{Z}<z^*} z[f_Z(z|X \geq x) - f_Z(z|X \leq x)]\mathrm{d}z + \\
&\int_{\mathcal{Z}\geq z^*} z[f_Z(z|X \geq x) - f_Z(z|X \leq x)]\mathrm{d}z \\
\geq &z^* \int_{\mathcal{Z}<z^*} [f_Z(z|X \geq x) - f_Z(z|X \leq x)]\mathrm{d}z + \\
&z^* \int_{\mathcal{Z}\geq z^*} [f_Z(z|X \geq x) - f_Z(z|X \leq x)]\mathrm{d}z \\
= &z^* \int_{\mathcal{Z}} [f_Z(z|X \geq x) - f_Z(z|X \leq x)]\mathrm{d}z = 0
\end{aligned} \tag{4}
$$

which means that $W(x) \geq 0$. This conclusion is easy to extend to the more general case that $X = m(Z) + g_X(U, \epsilon_X)$ with a monotone instrumental effect function $m(\cdot)$.

In the case that $\{Z, X\}$ follows a joint normal distribution that

$$
\begin{pmatrix} Z \\ X \end{pmatrix} \sim \mathcal{N}\left(\begin{pmatrix} \mu_Z \\ \mu_X \end{pmatrix}, \begin{pmatrix} \sigma_Z^2 & \sigma_{Z,X} \\ \sigma_{Z,X} & \sigma_X^2 \end{pmatrix} \right) \tag{5}
$$

which means

$$
\mathbb{E}(Z|X = x) = \mu_Z + \frac{\sigma_{Z,X}}{\sigma_X^2}(x - \mu_X)
$$

We have the weight function

$$
\begin{aligned}
W(s) = &\frac{1}{\sigma_{Z,X}} \left[\mathbb{E}(I\{X \geqslant s\}Z) - \mathbb{E}(I\{X \geqslant s\})\mathbb{E}(Z)\right] \\
= &\frac{1}{\sigma_{Z,X}} \left[\int_{[s,\infty)} f_X(x) \int z f_{Z|X}(z|x) dz dx - \int_{[s,\infty)} f_X(x) \int z f_Z(z) dz dx \right] \\
= &\frac{1}{\sigma_{Z,X}} \left[\int_{[s,\infty)} f_X(x) \left[\mathbb{E}(Z|X = x) - \mathbb{E}(Z)\right] dx \right] \\
= &\frac{1}{\sigma_{Z,X}} \left[\int_{[s,\infty)} f_X(x) \left[\frac{\sigma_{Z,X}}{\sigma_X^2}(x - \mu_X) \right] dx \right] \\
= &\frac{1}{\sigma_X^2} \int_{[s,\infty)} f_X(x)(x - \mu_X) dx \\
= &\frac{1}{\sqrt{2\pi}\sigma_X} \int_{[s,\infty]} \frac{1}{\sigma_X^2} \exp\left\{ -\frac{(x - \mu_X)^2}{2\sigma_X^2} \right\} (x - \mu_X) dx \\
= &\frac{1}{\sqrt{2\pi}\sigma_X} \left\{ -\exp\left(-\frac{(x - \mu_X)^2}{2\sigma_X^2} \right) \right\} \Bigg|_s^\infty = \frac{1}{\sqrt{2\pi}\sigma_X} \exp\left\{ -\frac{(s - \mu_X)^2}{2\sigma_X^2} \right\} = f_X(s)
\end{aligned} \tag{6}
$$

which means $W(s)$ is the marginal normal density of X. ■

Appendix E: Linearity testing

The null hypothesis of effect linearity implies that $h'(x)$ remains constant for all x, equaling the average treatment effect β. Denoting the stratum-specific genetic association estimators for the exposure and the outcome as $\hat{\alpha}_k$ and $\hat{\theta}_k$, respectively, we obtain the asymptotic result for each stratum:

$$\frac{\hat{\theta}_k}{\hat{\alpha}_k} \xrightarrow{p} \beta \qquad \text{for } s = 1, \ldots, K. \tag{7}$$

Since stratification results in multiple strata ($K > 1$), we can leverage over-identification or coherence approaches to test for effect linearity based on Equation (7) (Hansen, 1982). A common approach is to use Cochran's Q-based statistic:

$$Q(\beta) := \sum_{k=1}^{K} \left\{ \frac{\left(\hat{\theta}_k - \beta\hat{\alpha}_k\right)^2}{s.e.(\hat{\theta}_k)^2 + \beta^2 s.e.(\hat{\alpha}_k)^2} \right\}, \tag{8}$$

which is a simplified version (e.g., ignoring the correlation between $\hat{\theta}_k$ and $\hat{\alpha}_k$) commonly used in meta-analysis and Mendelian randomization (Hardy and Thompson, 1998; Greco M et al., 2015).

In practice, we compare the observed Q statistic, $Q(\hat{\beta})$, where $\hat{\beta} = \arg\min_\beta Q(\beta)$ or any fundamental IV estimator under the linearity assumption, to its approximated χ^2 distribution with degrees of freedom $K - 1$ under the null hypothesis. The Q statistic can also be interpreted as the maximal profile likelihood in an error-in-variable model that accounts for measurement error in α_k, treating α_k as a nuisance parameter (Zhao et al., 2020), or as the residual sum of squares from an inverse-variance weighted (IVW) regression of $\hat{\theta}_k$ on $\hat{\alpha}_k$ using second-order weights (Bowden et al., 2019). Rejection of the Q test provides evidence for effect nonlinearity.

Appendix F: Complicated model scenario and their model properties

In this section, we extend our analysis to a more complex model

$$Y = h(X, Z, \epsilon) + g(U, Z, \epsilon). \tag{9}$$

We assume that the instrument Z can be used for stratification, ensuring that Features 1 and 2 hold. Under this assumption, we construct the SoF model introduced before within each stratum. For reference, we define the simplest model, $Y = h(X) + U$, as **Scenario 0**. Below, we outline several complex model scenarios, each designed for specific objectives, along with illustrative examples.

Complex scenario 1. *When there exists exogenous effect modifiers such that*

$$Y = h(X, \epsilon) + g(U, \epsilon) \tag{10}$$

with regular conditions, all the ideas introduced above can be applicable to the objectives described by the mean effect function, $h(x) := \mathbb{E}_\epsilon(h(x, \epsilon))$, or the modifier-controlled effect function, $h(x, \epsilon^*)$, for a modifier layer ϵ^* when the modifier is measurable. For example, sex can be the measured modifier of the effect function, hence sex-strata may be first constructed for any downstream sex-specific nonlinear effect analysis. In addition, sex generally cannot be the downstream effect of the instrument and the confounder, hence selection on sex should not induce collider bias.

Complex scenario 2. *In the case that there exists the additive instrumental direct effect on the outcome (one typical case that violation of the exclusion restriction), in the sense that*

$$Y = h(X, \epsilon) + g(Z, U, \epsilon) \tag{11}$$

if we have the decomposition that $g(Z, U, \epsilon) = g_1(Z, \epsilon) + g_2(U, \epsilon)$ so that the instrumental direct effect is not modified by confounders, one can still utilize the stratification and SoF model to test the effect linearity via testing the constant value among the stratum-specific IV estimates. The decomposition property is a quite common condition used in invalid IV models, like Bowden et al. (2015); Tchetgen Tchetgen et al. (2021). The decomposition property allows that the linearity test is now robust to certain invalid IV cases, and also indicates that certain confounders in some applications can serve as the instrument for linearity testing. For example, in the exposure scenario where one believes the rank-preserving assumption between age and exposure is plausible and the age also has a direct effect on the outcome, one may still able to treat age as the 'instrument' to test the linear effect of the exposure on the outcome. Similarly in Mendelian randomization, some genes may have the direct effect on the outcome rather than through the exposure, which in genetics is called horizontal pleiotropy (Paaby and Rockman, 2013); if one believes the genetic direct effect is not modified by the confounder, they are still valid for effect linearity testing even though they are invalid in IV context.

Complex scenario 3. *In the case that the instrument modifies the effect of the exposure on the outcome (i.e. another typical case that violation of the exclusion restriction), in the sense that*

$$Y = h(X, Z, \epsilon) + g(U, \epsilon) \tag{12}$$

if we have the factorization that $h(X, Z, \epsilon) = h_0(X, \epsilon)f(Z, \epsilon)$, one can still utilize the stratification and SoF model to test the effect linearity regarding $h_0(\cdot)$ via testing the constant value among the stratum-specific IV estimates (details see the previous appendix). Note that this conclusion can be combined with the further decomposition scenario that $g(Z, U, \epsilon) = g_1(Z, \epsilon) + g_2(U, \epsilon)$. For example, in some applications where the age (treated as an invalid instrument) has both the additive direct effect and the modified effect on the outcome, the linearity of the exposure effect can still be tested, given that the stratification assumption and the decomposition/factorization model assumption are plausible.

We prove the properties with a more complex model. Since the variable ϵ is exogenous and any stratification based on (Z, X) will not change the distribution of ϵ, according to the previous appendix we will drop it for notation simplification.

Given the model with decomposition

$$Y = h(X) + g_1(Z) + g_2(U) \tag{13}$$

Assume that stratification can be implemented to (Z, X) to construct K strata, and the stratum-specific exchangeability $Z \perp\!\!\!\perp U | S$ hold. The stratum-specific IV estimator has the asymptotic value

$$\begin{aligned}
\frac{Cov_k(Z, Y)}{Cov_k(Z, X)} &= \frac{Cov_k(Z, \int_{\mathcal{X}} h'(x) I\{X \geq x\} \mathrm{d}x + g_1(Z))}{Cov_k(Z, X)} \\
&= \int_{\mathcal{X}} h'(x) \frac{Cov_k(Z, I\{X \geq x\})}{Cov_k(Z, X)} \mathrm{d}x + \frac{Cov_k(Z, g_1(Z))}{Cov_k(Z, X)} \\
&= \int_{\mathcal{X}} h'(x) W_k(x) \mathrm{d}x + \frac{c_0}{Cov_k(Z, X)}
\end{aligned} \tag{14}$$

where $Cov_k(Z, g_1(Z)) = c_0$ is a constant regardless of strata index due to the stratification property that the distribution of the instrument is constant across strata (this also indicates that $Var_k(Z)$ is constant over s). Therefore, under the null hypothesis of effect linearity that $h'(x) = c_1$, we have the condition, similar to meta-analysis

$$\hat{\theta}_k = c_0 + c_1 \alpha_k + \epsilon_k \qquad \text{with } \epsilon_k \sim \mathcal{N}(0, s.e.(\hat{\theta}_k)^2) \tag{15}$$

With the over-identification idea, we can test the model via either the simplified form treating $\hat{\alpha}_k = \alpha_k$ (i.e. no measured error) or consider the uncertainty of $\{\hat{\alpha}_k\}$ under errors-in-variable model

framework with the Cochran's Q test statistic

$$Q(c_0, c_1) = \sum_{s=1}^{K} \left\{ \frac{\left(\hat{\theta}_k - c_0 - c_1\hat{\alpha}_k\right)^2}{s.e.(\hat{\theta}_k)^2 + c_1^2 s.e.(\hat{\alpha}_k)^2} \right\}, \tag{16}$$

the Q test statistic $Q(\hat{c}_0, \hat{c}_1)$, where $(\hat{c}_0, \hat{c}_1) = \arg\min_{(c_0,c_1)} Q(c_0, c_1)$ under null has the approximated Chi-squared distribution with degree-of-freedom $K - 2$.

Given the model with the factorization

$$Y = h(X)f(Z) + g(U) \tag{17}$$

We have

$$\begin{aligned}
\theta_k = \frac{Cov_k(Z, Y)}{Var_k(Z)} &= \frac{\int_{\mathcal{X}} Cov_k(Z, f(Z)h'(x)I\{X \geq x\})\mathrm{d}x}{Var_k(Z)} \\
&= \beta\frac{\int_{\mathcal{X}} Cov_k(Z, f(Z)I\{X \geq x\})\mathrm{d}x}{Var_k(Z)} \quad \text{under the null that } h'(x) \equiv \beta \\
&= \beta\frac{Cov_k(Z, f(Z)(X - l))}{Var_k(Z)} \quad \text{where } X \geq l \text{ surely} \\
&= \beta\frac{Cov_k(Z, f(Z)X)}{Var_k(Z)} - l\,\beta\frac{Cov_k(Z, f(Z))}{Var_k(Z)} \\
&= \beta\frac{Cov_k(Z, f(Z)X)}{Var_k(Z)} - l\,\beta\,c
\end{aligned} \tag{18}$$

If one wish to make assumption on $f(Z)$ or for binary instrument Z such that $f(Z) = 1 + \tau Z$ nonparametrically, the equation can be further expressed as

$$\begin{aligned}
\theta_k = \frac{Cov_k(Z, Y)}{Var_k(Z)} &= \beta\frac{Cov_k(Z, X + \tau ZX)}{Var_k(Z)} - l\,\beta\,c \\
&= \beta\frac{Cov_k(Z, X)}{Var_k(Z)} + \beta\,\tau\frac{Cov_k(Z, ZX)}{Var_k(Z)} - l\,\beta\,c \\
&= \beta\alpha_k + c_1\gamma_k + c_0
\end{aligned} \tag{19}$$

where $c_0 = l\,\beta\,c$, $c_1 = \beta\,\tau$ and γ_k is the s-th stratum-specific instrumental association with ZX. Hence, the effect linearity test using Cocharan's Q is based on the Q statistic

$$Q(c_0, c_1, \beta) = \sum_{s=1}^{K} \left\{ \frac{\left(\hat{\theta}_k - c_0 - c_1\hat{\gamma}_k - \beta\hat{\alpha}_k\right)^2}{s.e.(\hat{\theta}_k)^2 + c_1^2 s.e.(\hat{\gamma}_k)^2 + \beta^2 s.e.(\hat{\alpha}_k)^2} \right\}, \tag{20}$$

the Q test statistic $Q(\hat{c}_0, \hat{c}_1, \hat{\beta})$, where $(\hat{c}_0, \hat{c}_1, \hat{\beta}) = \arg\min_{(c_0,c_1,\beta)} Q(c_0, c_1, \beta)$ under null has the approximated Chi-squared distribution with degree-of-freedom $K - 3$.

Appendix G: Complete algorithm of the SSS framework

Algorithm 3: The three 'S' framework (SSS) for nonlinear effect analysis

Input: individual-level data $(Z_i, X_i, Y_i)_{i=1,\ldots,n}$
Output: results for Objective 1-4 (e.g. estimated effect function, the predicted change-point)

$K \leftarrow$ the strata number
$P \leftarrow$ the basis-functions number in parametric fitting or quantile number in the change-point model
$L \leftarrow$ the iteration number in SuSiE fitting

First 'S': Stratification
$\mathcal{S} \leftarrow$ strata set (size of K) by the doubly-ranked or residual stratification by Algorithm 1 or 2
for *each stratum* $k \leftarrow 1$ **to** K **do**
    Obtain the point summary statistics from the k-th strata:
    - estimated instrumental association with X and its s.e. : $\hat{\alpha}_k$, $s.e.(\hat{\alpha}_k)$
    - estimated instrumental association with Y and its s.e. : $\hat{\theta}_k$, $s.e.(\hat{\theta}_k)$

    Obtain the weight function from the k-th strata:

$$\hat{W}_k(x) = \frac{\widehat{Cov}_k(Z, I\{X \geq x\})}{\widehat{Cov}_k(Z, X)}$$

(Optional) linearity testing using $\{\hat{\alpha}_k,\ s.e.(\hat{\alpha}_k), \hat{\theta}_k,\ s.e.(\hat{\theta}_k)\}$, e.g., via Q test in Eq (8)

Second 'S': Scalar-on-function or scalar-on-scalar regression
if *basis function assumption* $\{\phi_l(x)\}$ *made* **then**
    construct the covariates: $\langle \phi_p, \hat{W}_k \rangle$ for all p and k
    construct the scalar-on-function or scalar-on-scalar regression using the covariates above and $\{\hat{\alpha}_k,\ s.e.(\hat{\alpha}_k), \hat{\theta}_k,\ s.e.(\hat{\theta}_k)\}_{k=1,\ldots,K}$
    if *regularization required* **then**
        Determine the tuning parameter λ, e.g., using GCV
    Perform parametric fitting
    Return: the fitted effect function $\hat{h}(x)$ and its pointwise confidence interval

Third 'S': SuSiE (only with scalar-on-function regression)
Construct the covariates: $\langle I\{x \geq t_p\}, \hat{W}_k \rangle$ for all p and k
Build the change-point model (piecewise constant effect model)
Conduct sum of single effect fitting, and obtain
- $\boldsymbol{\pi}^*$, the matrix posterior inclusion probabilities
- $\boldsymbol{\mu}^*$, the matrix of posterior mean
- $\boldsymbol{\sigma}^*$, the matrix of the posterior standard deviation

Return: the predicted value or/and credible set of the changepoint based on $\boldsymbol{\pi}^*$
Return: the predicted effect function $\hat{h}(x)$ and its pointwise credibility set based on $\boldsymbol{\pi}^*, \boldsymbol{\mu}^*, \boldsymbol{\sigma}^*$

Appendix H: Supplementary simulation

We repeat the simulation from Part I using a nonlinear effect function. The setup remains similar, and we consider the following scenarios for the instrument type and outcome structural models with a nonlinear effect:

$$\begin{array}{lll} \text{Scenario 1:} & Z \sim \text{Bernoulli}(0.5) - 0.5 & Y = I\{X > 0\} + U + \epsilon_Y \\ \text{Scenario 2:} & Z \sim \text{Bernoulli}(0.5) - 0.5 & Y = I\{X > 0\} + |U| + \epsilon_X^2 + 2|U||\epsilon_X| + \epsilon_Y \\ \text{Scenario 3:} & Z \sim \mathcal{N}(0,1) & Y = I\{X > 0\} + U + \epsilon_Y \\ \text{Scenario 4:} & Z \sim \mathcal{N}(0,1) & Y = I\{X > 0\} + |U| + \epsilon_X^2 + 2|U||\epsilon_X| + \epsilon_Y \end{array} \tag{21}$$

where the effect shape function is defined as $h(x) = I\{x > 0\}$, which is nonlinear with a change-point at $x = 0$. We consider the same methods (M1–M5) as in the main text but exclude M6 (DeepIV) and M7 (KernelIV) in this supplementary simulation due to their significantly long runtime. For the originally oracle methods (M1–M3), we continue using a polynomial basis function of up to the second order because such a polynomial is a common choice in practice. While it may not perfectly capture the nonlinear effect $h(x) = I\{x > 0\}$, its second-order property allows it to approximate the shape reasonably well across different exposure levels.

The MSE results are provided in Table 1. The 'oracle' methods (M1–M3) no longer achieve the best MSE performance in many quantile cases, particularly for quantiles below 50%, where the effect is zero. In contrast, the SSS method (M5) demonstrates the best MSE performance in more scenarios, despite having greater estimation uncertainty due to its model flexibility. This can be attributed to the SSS method's ability to accommodate change-point effect patterns, whereas other methods rely solely on polynomial approximations of the underlying effect shape. When the polynomial order is low, as in M1–M3, it often fails to accurately approximate the effect shape in lower exposure quantiles. This may explain why many nonlinear methods tend to estimate a J-shaped effect function, even in cases where the true effect remains zero until a certain change-point.

We also consider another nonlinear effect shape case, so that

$$\begin{array}{lll} \text{Scenario 1:} & Z \sim \text{Bernoulli}(0.5) - 0.5 & Y = \exp\{0.5X\} + U + \epsilon_Y \\ \text{Scenario 2:} & Z \sim \text{Bernoulli}(0.5) - 0.5 & Y = \exp\{0.5X\} + |U| + \epsilon_X^2 + 2|U||\epsilon_X| + \epsilon_Y \\ \text{Scenario 3:} & Z \sim \mathcal{N}(0,1) & Y = \exp\{0.5X\} + U + \epsilon_Y \\ \text{Scenario 4:} & Z \sim \mathcal{N}(0,1) & Y = \exp\{0.5X\} + |U| + \epsilon_X^2 + 2|U||\epsilon_X| + \epsilon_Y \end{array} \tag{22}$$

The MSE results are provided in Table 2. Due to the good approximation ability of the second-order polynomial to the underlying exponential effect, the MSE results for M1–M4 are similar to those in the main text using a linear effect. The main difference is that the SSS method (M5) exhibits better MSE performance in many cases.

	Scenario 1 (binary IV, simple confounding)					Scenario 3 (continuous IV, simple confounding)				
	M1	M2	M3	M4	M5	M1	M2	M3	M4	M5
10%	0.519	0.460	0.599	0.720	**0.125**	0.250	0.123	0.281	0.292	**0.042**
30%	0.130	0.105	0.138	0.112	**0.055**	0.094	0.063	0.098	0.082	**0.025**
50%	0	0	0	0	0	0	0	0	0	0
70%	0.182	**0.140**	0.144	0.308	0.182	0.101	0.071	0.100	0.135	**0.069**
90%	0.707	**0.449**	0.631	1.584	0.892	0.276	**0.118**	0.299	0.458	0.227
	Scenario 2 (binary IV, complex confounding)					Scenario 4 (continuous IV, complex confounding)				
	M1	M2	M3	M4	M5	M1	M2	M3	M4	M5
10%	3.856	27.509	1.387	1.163	**0.189**	3.180	26.206	0.626	0.636	**0.146**
30%	0.154	0.771	0.231	0.125	**0.073**	**0.036**	0.504	0.134	0.100	0.058
50%	0	0	0	0	0	0	0	0	0	0
70%	**0.157**	0.838	0.252	0.498	0.305	**0.035**	0.535	0.127	0.325	0.182
90%	4.024	28.823	**1.658**	3.046	1.765	3.125	26.805	**0.549**	1.625	0.815

Table 1: The MSE results of the effect function over several exposure quantiles across multiple nonlinear IV methods (denoted by M1-M5) under four different model scenarios in 1000 simulations. The underlying effect is $h(x) = I\{x > 0\}$. The objective of the effect function is defined to be zero when the exposure level is 0, corresponding to the 50% quantile in each scenario so the MSE is 0. For each scenario and quantile value, the minimal MSE value is highlighted. M1: Oracle control function. M2: Oracle IV-regression. M3: Stratification with oracle SoS regression. M4: PolyMR. M5: SSS. The oracle methods use the basis function as the polynomial up to second order in fitting.

	Scenario 1 (binary IV, simple confounding)					Scenario 3 (continuous IV, simple confounding)				
	M1	M2	M3	M4	M5	M1	M2	M3	M4	M5
10%	0.421	0.417	0.428	0.659	**0.223**	**0.067**	0.119	0.099	0.209	0.151
30%	0.071	0.071	0.061	0.103	**0.060**	**0.016**	0.025	0.017	0.038	0.038
50%	0	0	0	0	0	0	0	0	0	0
70%	0.075	0.073	**0.065**	0.122	0.084	0.024	0.028	**0.021**	0.039	0.038
90%	0.427	0.417	**0.459**	0.939	0.583	**0.111**	0.131	0.122	0.250	0.159
	Scenario 2 (binary IV, complex confounding)					Scenario 4 (continuous IV, complex confounding)				
	M1	M2	M3	M4	M5	M1	M2	M3	M4	M5
10%	5.429	25.954	1.450	1.376	**0.294**	4.743	24.875	0.456	0.635	**0.221**
30%	0.232	0.877	0.175	0.195	**0.084**	0.113	0.659	**0.055**	0.107	0.057
50%	0	0	0	0	0	0	0	0	0	0
70%	0.396	1.289	0.222	0.257	**0.159**	0.259	1.033	**0.059**	0.124	0.082
90%	6.809	29.729	1.945	2.384	**1.400**	5.856	28.062	**0.468**	0.910	0.526

Table 2: The MSE results of the effect function over several exposure quantiles across multiple nonlinear IV methods (denoted by M1-M5) under four different model scenarios in 1000 simulations. The underlying effect is $h(x) = \exp\{0.5x\}$.

Appendix I: UK Biobank real application with females

We replicated the real-data analysis from the main text in the female sample to investigate the nonlinear effect of alcohol intake on SBP. The genetic association with alcohol consumption is known to differ between males and females (e.g., cultural factors may reduce drinking among females), so the stratification assumptions may be violated in a mixed-sex sample. In addition, the causal effect of alcohol on many traits may be heterogeneous across sexes since women and men may metabolize alcohol differently (Graham et al., 1998). Therefore, this analysis should not be viewed as a strict replication of the male-only analysis presented in the main text.

We used the same setup as in the main text. Specifically, we analyzed 150,600 female individuals of European ancestry and for each participant we constructed a weighted genetic score from the same 93 variants used in the male text. We adopted doubly-ranked stratification, forming 10 strata for presenting stratum-specific results and 100 strata for estimation. We also treated age as a negative control outcome. For each stratum, we obtained point IV estimates and the corresponding functional weight functions. We then built a scalar-on-function regression and considered 95 change-point candidates corresponding to the lower 95% quantiles of the original alcohol-intake distribution in females, $\{\, t_p := \hat{F}_X^{-1}(p/100) \,\}_{p=0,1,\ldots,95}$.. We fitted the regression using SuSiE with the iteration number $L = 10$ and obtained posterior inclusion probabilities for the change-points. We further computed the pointwise estimate and the 95% credible interval for the effect shape function $h(x) := \mathbb{E}\{Y(x) - Y(0)\}$. Complete results are shown in Figure 3. Overall, the findings in females mirror those in males, supporting a positive effect of alcohol intake on SBP, with a comparable credible-interval range for the effect change-point location: $(0, 3.271)$ in females versus $(0, 3.286)$ in males under the same setting.

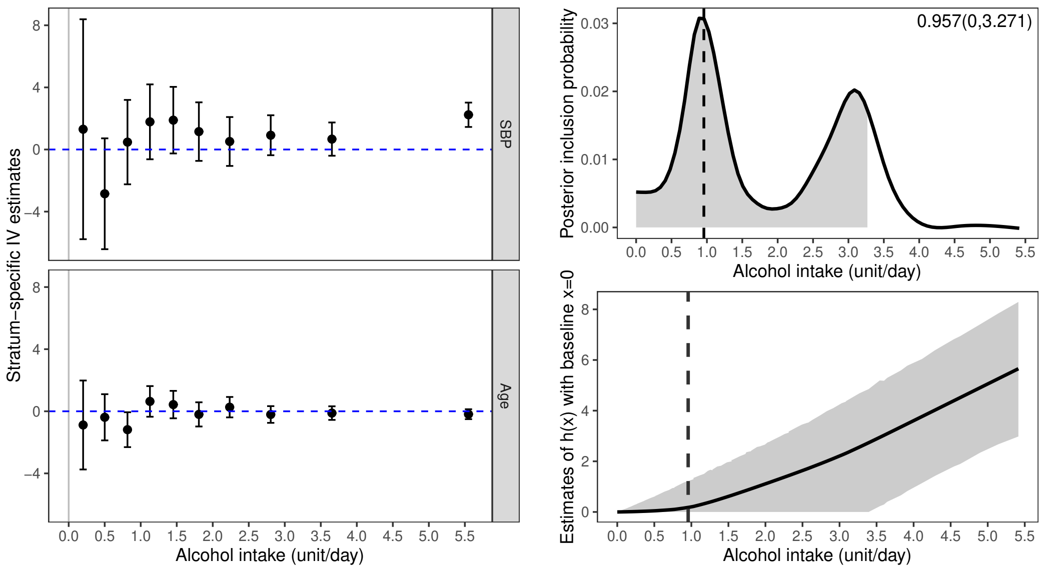

Figure 3: Left: Stratum-specific IV estimates for SBP and Age, obtained using doubly-ranked stratification with 10 strata (shown here for clarity of presentation).
Right: Change-point and effect-function inference results from the "SSS" framework, using doubly-ranked stratification with 100 strata and Sum-of-Single-Effect (SuSiE) estimation. Upper panel: Posterior inclusion probabilities of change-points and the 95% credible interval for the change-point location. Lower panel: Estimated effect function with its 95% credible interval. The vertical dashed line marks the posterior mode of the change-point. Only one change-point was detected.

Appendix J: Supplementary results of the real application

We conducted effect shape estimation for the real application using other common choices for the number of strata, namely $K = 10$ and $K = 50$. The estimation results are presented in Figures 4 and 5. The results are highly similar for both choices of K, indicating that our findings are insensitive to the number of strata.

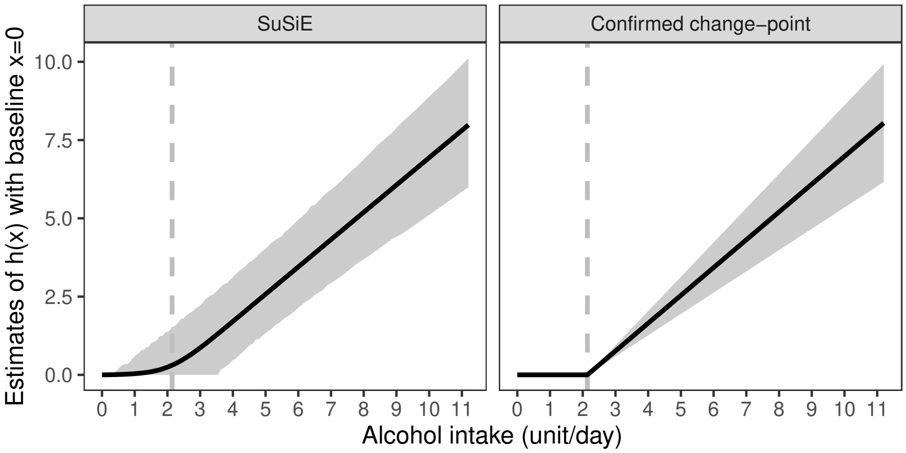

Figure 4: The estimate of the effect shape $h(x)$ using two fitting strategies with the number of strata $\boldsymbol{K = 10}$: (left panel) SuSiE fitting where the point estimate $\hat{h}(x)$ is the posterior mean and the interval estimate is the 95% credible interval; and (right panel) the parametric fitting with the confirmed change-point ($x = 2.142$, the dashed vertical line, using the posterior mode) for basis functions, and the interval estimate is the 95% confidence interval. The effect shape $h(x)$ is defined with the baseline value $x = 0$ so that $h(0) = 0$.

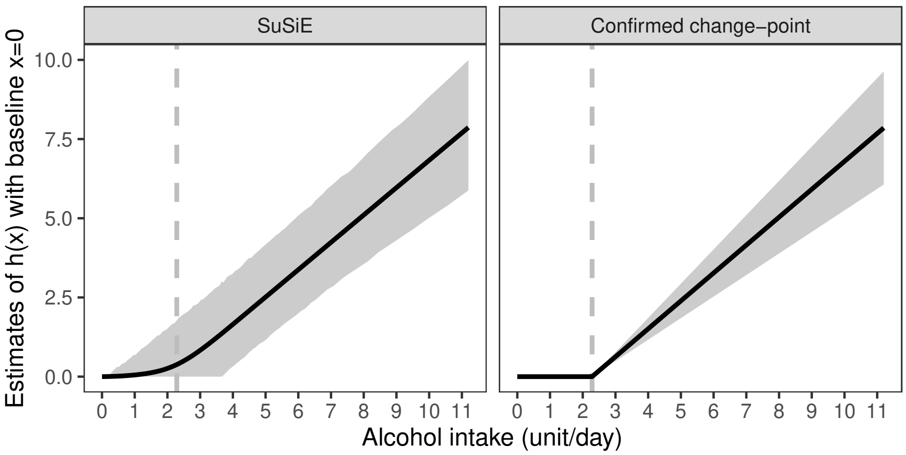

Figure 5: The estimate of the effect shape $h(x)$ using two fitting strategies with the number of strata $\boldsymbol{K = 50}$: (left panel) SuSiE fitting where the point estimate $\hat{h}(x)$ is the posterior mean and the interval estimate is the 95% credible interval; and (right panel) the parametric fitting with the confirmed change-point ($x = 2.286$, the dashed vertical line, using the posterior mode) for basis functions, and the interval estimate is the 95% confidence interval. The effect shape $h(x)$ is defined with the baseline value $x = 0$ so that $h(0) = 0$.

References

Joshua D Angrist and Alan B Krueger. Does compulsory school attendance affect schooling and earnings? *The Quarterly Journal of Economics*, 106(4):979–1014, 1991.

Jack Bowden, George Davey Smith, and Stephen Burgess. Mendelian randomization with invalid instruments: effect estimation and bias detection through egger regression. *International journal of epidemiology*, 44(2):512–525, 2015.

Jack Bowden, Fabiola Del Greco M, Cosetta Minelli, Qingyuan Zhao, Debbie A Lawlor, Nuala A Sheehan, John Thompson, and George Davey Smith. Improving the accuracy of two-sample summary-data mendelian randomization: moving beyond the nome assumption. *International journal of epidemiology*, 48(3):728–742, 2019.

Kathryn Graham, Richard Wilsnack, Deborah Dawson, and Nancy Vogeltanz. Should alcohol consumption measures be adjusted for gender differences? *Addiction*, 93(8):1137–1147, 1998.

Fabiola Del Greco M, Cosetta Minelli, Nuala A Sheehan, and John R Thompson. Detecting pleiotropy in mendelian randomisation studies with summary data and a continuous outcome. *Statistics in medicine*, 34(21):2926–2940, 2015.

Lars Peter Hansen. Large sample properties of generalized method of moments estimators. *Econometrica: Journal of the econometric society*, pages 1029–1054, 1982.

Rebecca J Hardy and Simon G Thompson. Detecting and describing heterogeneity in meta-analysis. *Statistics in medicine*, 17(8):841–856, 1998.

Jeffrey R Kling. Incarceration length, employment, and earnings. *American Economic Review*, 96 (3):863–876, 2006.

Annalise B Paaby and Matthew V Rockman. The many faces of pleiotropy. *Trends in genetics*, 29 (2):66–73, 2013.

Eric Tchetgen Tchetgen, BaoLuo Sun, and Stefan Walter. The genius approach to robust mendelian randomization inference. *Statistical Science*, 36(3):443–464, 2021.

Haodong Tian, Amy M Mason, Cunhao Liu, and Stephen Burgess. Relaxing parametric assumptions for non-linear mendelian randomization using a doubly-ranked stratification method. *PLoS genetics*, 19(6):e1010823, 2023.

Qingyuan Zhao, Jingshu Wang, Gibran Hemani, Jack Bowden, and Dylan S Small. Statistical inference in two-sample summary-data mendelian randomization using robust adjusted profile score. *The Annals of Statistics*, 48(3):1742–1769, 2020.
